# Rapid Characterization and Identification of Non-Diterpenoid Constituents in *Tinospora sinensis* by HPLC-LTQ-Orbitrap MS^n^

**DOI:** 10.3390/molecules23020274

**Published:** 2018-01-28

**Authors:** Qi-Shu Jiao, Lu-Lu Xu, Jia-Yu Zhang, Zi-Jian Wang, Yan-Yan Jiang, Bin Liu

**Affiliations:** 1 School of Chinese Pharmacy, Beijing University of Chinese Medicine, Beijing 102488, China; qs_jiao1108@bucm.edu.cn (Q.-S.J.); xll@bucm.edu.cn (L.-L.X.); jyyjm1129@163.com (Y.-Y.J.); 2 Beijing Research Institution of Chinese Medicine, Beijing University of Chinese Medicine, Beijing 100029, China; zhangjiayu0615@163.com (J.-Y.Z.); helloffiresilver@gmail.com (Z.-J.W.)

**Keywords:** HPLC-LTQ-Orbitrap, non-diterpenoids, fragmentation pathway, *Tinospora sinensis*

## Abstract

*Tinospora sinensis*, a kind of Chinese folk medicine, has functions of harmonizing qi and blood, dredging the channels and collaterals, calming and soothing the nerves. In the present study, a method based on high-performance liquid chromatography coupled with linear ion trap-Orbitrap mass spectrometry (HPLC-LTQ-Orbitrap) was developed for the systematical characterization of the non-diterpenoid constituents which possessed remarkable biological activities in *T*. *sinensis,* like anti-tumor, anti-inflammatory, hypoglycemic activity and immunomodulatory activity. Based on the accurate mass measurement (<5 ppm), retention times and MS fragmentation ions, 60 non-diterpenoid constituents were unambiguously or tentatively characterized from *T. sinensis* extract, including 27 alkaloids, 23 phenylpropanoids, seven sesquiterpenoids and three other constituents. Among them, 13 compounds were tentatively identified as new compounds. Finally, three of the non-diterpenoid constituents were purified and identified, which further confirmed the validity of the results. This study demonstrated that the HPLC-LTQ-Orbitrap MS^n^ platform was a useful and efficient analytical tool to screen and identify constituents in natural medicine.

## 1. Introduction

*Tinospora sinensis* is derived from the dried stems of *Tinospora sinensis* (Lour.) Merr. (family Menispermaceae), which was officially documented in the *Chinese Pharmacopoeia* (2015) with the name Kuanjinteng [1]. As a kind of folk medicine which is mainly distributed in China, India and Southeast Asia, *T. sinensis* is commonly employed to treat various diseases. For instance, Tibetan medicine thought that *T. sinensis* could be used to treat rheumatoid arthritis, while Indian Ayurveda usually employed this ethnic drug in treatment for diabetes [2]. Pharmacological studies and clinical practice have demonstrated that the extracts of *T. sinensis* possessed various biological activities, including anti-inflammatory, anti-oxidative, anti-radiant, insecticidal and immunosuppressive effects [3,4,5,6].

*T. sinensis* has complicated chemical composition, including diterpenoids, alkaloids, phenylpropanoids, sesquiterpenes, triterpenoids, sterols, amino acids, and so on. Among them, diterpenoids are considered as the most abundant constituents. In previous work, we have systematic reported of diterpenoids in *T. sinensis*. A total of 63 diterpenoids were preliminarily identified, including 10 diterpenoid aglycones and 53 diterpenoid glycosides [7]. However, some non-diterpenoid constituents show good pharmacological activities which are probably closely related to its traditional efficacy. For example, two carboxylic acid esters isolated from *T. sinensis* showed significant PTP1B inhibitory activity in vitro, which represented a novel strategy for the treatment of type II diabetes [8]. Diosgenin isolated from *T. sinensis* showed good anti-inflammatory activity in carrageenan induced inflammation (paw edema) rodent model [9]. Three isoquinoline alkaloids isolated from *T. cordifolia* stem, named jatrorrhizine, palmatine and magnoflorine, were proved to possess the potential inhibitory effect on *α*-glucosidase in vitro and in vivo [10]. *Tans*-syringin, a typical phenylpropanoid glycoside which was abundant in *T. sinensis*, showed various activities of anti-tumor, anti-inflammatory, and hypoglycemic activities [11,12,13]. In addition, two sesquiterpenes of tinocordiside and 11-hydroxymustakone isolated from *Tinospora cordifolia* showed significant immunomodulatory activity [14]. Therefore, in order to comprehensively expound the material foundation of efficacy of *T. sinensis*, we propose a strategy to screen and identify the non-diterpenoid constituents in this herb.

As a powerful tool with the high resolution and excellent sensitivity to analyze multi-constituents in complex matrices, HPLC-ESI-MS^n^ has been employed for characterization of phytochemical compounds in many areas of food and biological analyses [15,16]. The hybrid linear ion trap-Orbitrap mass spectrometer (LTQ-Orbitrap) was characterized by the higher mass resolution and mass accuracy (within 5 ppm) of orbitrap, MS^n^ scanning function, and high trapping capacity of the linear ion trap [17,18]. Orbitrap allows the potent detection of a great deal of chemical constituents of similar accurate mass with high confidence of compounds identification, especially combined with retention times and the use of mass spectral libraries constructed with authentic standards [19]. These advantages facilitate to rapidly identify and characterize of multiple constituents in TCMs. In general, compounds with the same carbon skeleton usually have similar fragmentation pathway and characteristic product ions in collision-induced dissociation (CID) mode, so that this method also could be employed to identify novel constituents in TCMs [20]. In this study, a method with HPLC-LTQ-Orbitrap was established to comprehensively analyze the non-diterpenoid constituents in *T. sinensis*.

## 2. Results and Discussion

### 2.1. Identification of the Constituents by HPLC-LTQ-Orbitrap MS^n^

**Figure 1:**
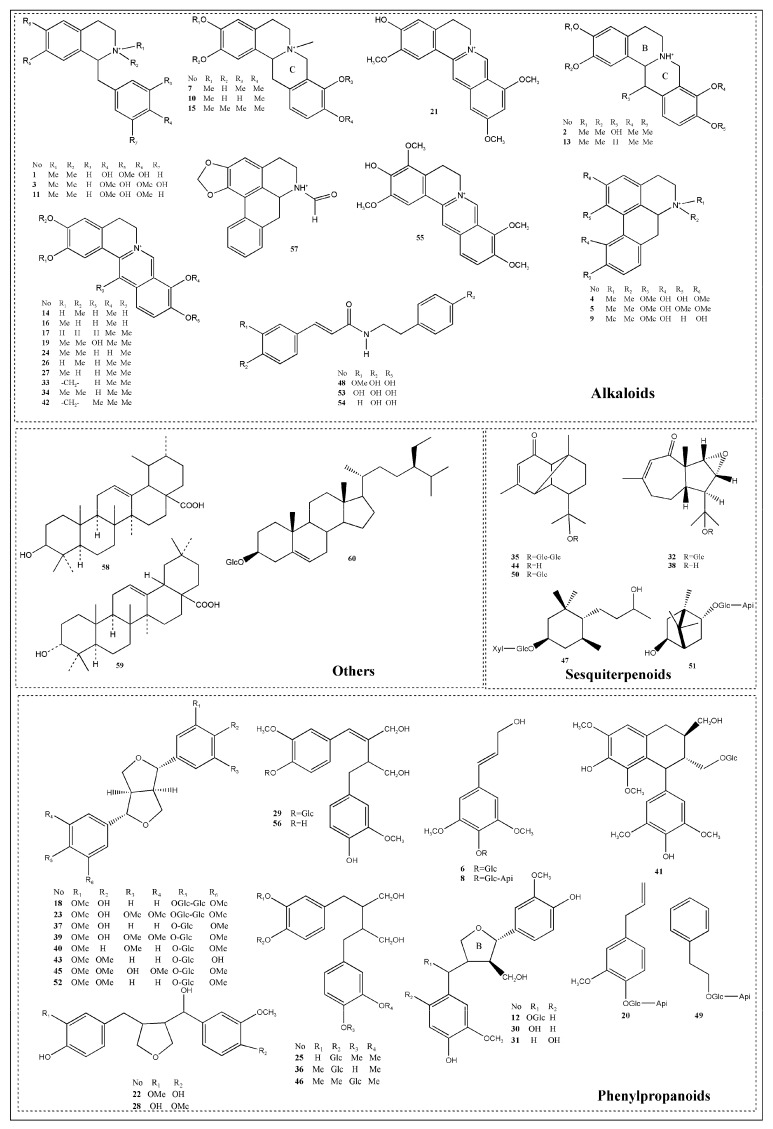
The structures of compounds identified in *T. sinensis.*

**Figure 2:**
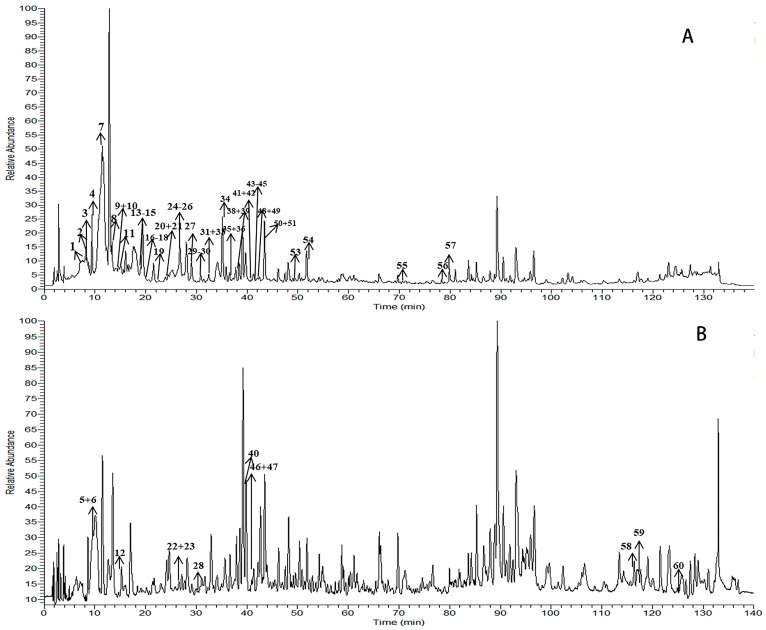
TIC chromatogram of *T. sinensis*: (**A**) positive ion mode; (**B**) negative ion mode.

In order to get adequate structural information of the chemical constituents in *T. Sinensis* and reveal as many chemical compounds as possible, both positive and negative modes were employed for the comprehensive analysis. For the available standard compounds, these compounds were identified by comparing retention time (*t*_R_) and/or accurate mass. For the standard unavailable compounds, the molecular formula of which were confirmed by compared with the HRMS molecular formula database built in-home, the high-accuracy protonated precursors with an error less than 5 ppm and related literatures. In the present study, a total of 60 compounds (Table 1, Figure 1) were identified or tentatively identified from *T. Sinensis* extract, including 27 alkaloids, 23 phenylpropanoids, seven sesquiterpenoids and three others. A typical total ion chromatogram (TIC) of *T. sinensis* in positive and negative ion mode is presented in Figure 2.

#### 2.1.1. Structural Characterization and Identification of Alkaloids

Compound **33** produced a [M]^+^ ion at *m*/*z* 336.12317 (C_20_H_18_NO_4_, mass error = 0.135 ppm). It yielded a series of ions at *m*/*z* 321 [M − CH_3_]^+^, *m*/*z* 320 [M − CH_3_ − H]^+^ and 306 [M − CH_3_ − CH_3_]^+^, suggesting the presence of adjacent methoxyl groups. The product ion at *m*/*z* 320 also generated the predominant produce ions at *m*/*z* 292 [M − CH_3_ − H − CO]^+^. In addition, the [M − 2H]^+^ ion at *m*/*z* 334 suggested the presence of C5–C6 carbon-carbon single bonds, which might due to a more stable π-conjugated system was formed by the losses of two hydrogen. By comparison with reference standard, compound **33** was predicatively deduced as berberine.

Compounds **21**, **24**, **26** and **27** generated their [M]^+^ ions at *m*/*z* 338.13861 (mass error = −0.075 ppm), *m*/*z* 338.13837 (mass error = −0.315 ppm), *m*/*z* 338.13834 (mass error = −0.345 ppm) and *m*/*z* 338.13843 (mass error = −0.255 ppm) (C_20_H_20_NO_4_), respectively. After the CID cleavage, all of them produced [M − CH_3_]^+^, [M − CH_3_ − CH_3_]^+^, [M − CH_3_ − CO]^+^ and [M − CH_4_ − CO]^+^ ions at *m*/*z* 323, *m*/*z* 308, *m*/*z* 295 and *m*/*z* 294, respectively. The *m*/*z* 320 [M − H_2_O]^+^ ions of compounds **21** and **26** suggested the presence of C-3 hydroxyl groups. Moreover, the [M − CH_3_ − H]^+^ ions of compounds **26** and **27** at *m*/*z* 322 suggested the presence of C9–C10 methoxyl groups. Therefore, combined with bibliography data and fragmentation pathways, these four compounds were tentatively identified as 3-hydroxy-2,9,11-tri-methoxy-5,6-dihydroisoquino[3,2-*α*]isoquinolinylium, palmaturbine, jatrorrhizine and columbamine, respectively [5,21].

Compound **34** showed the [M]^+^ ion at *m*/*z* 352.15390 (C_21_H_22_NO_4_, mass error = −0.435 ppm). In the MS^2^ spectrum, *m*/*z* 336 [M − CH_3_ − H]^+^ was identified as the base peak. Other product ions like *m*/*z* 337 [M − CH_3_]^+^, *m*/*z* 322 [M − 2CH_3_]^+^ and *m*/*z* 308 [M − CH_3_ − H − CO]^+^ were also observed. By comparison with the literature data, compound **34** was tentatively identified as palmatine [22].

**Scheme 1:**
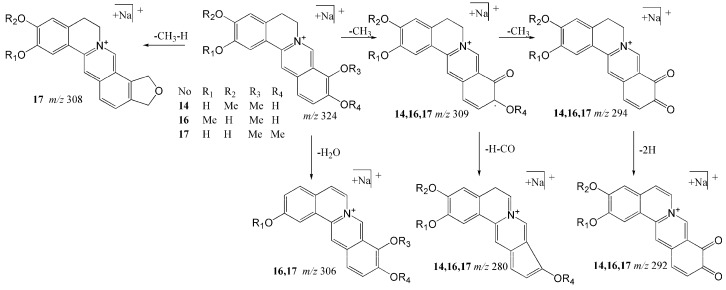
The proposed fragmentation pathway of stepharanine, dehydrodiscretamine and demethyleneberberine.

Compounds **14**, **16** and **17** gave their [M]^+^ ions at *m*/*z* 324.12292 (mass error = −0.115 ppm), *m*/*z* 324.12311 (mass error = 0.075 ppm) and *m*/*z* 324.12277 (mass error = −0.137 ppm) (C_19_H_18_NO_4_), respectively. They all produced the [M − CH_3_]^+^, [M − 2CH_3_]^+^, [M − 2CH_3_ − 2H]^+^ and [M − CH_3_ − H − CO]^+^ ions at *m*/*z* 309, *m*/*z* 294, *m*/*z* 292 and *m*/*z* 280, respectively. Besides, the [M − H_2_O]^+^ ions at *m*/*z* 306 of compounds **16** and **17** suggested that the presence of C-3 hydroxyl group. In addition, compound **17** could generate [M − CH_3_ − H]^+^ at *m*/*z* 308, suggesting that there were C9–C10 methoxyl groups at the skeleton [23]. The proposed fragmentation pathway of compound **14**, **16** and **17** are shown in Scheme 1. Therefore, compounds **14**, **16** and **17** were tentatively ascertained as stepharanine, dehydrodiscretamine and demethyleneberberine.

Compounds **19** and **55** yielded their quasi-molecular ions [M]^+^ at *m*/*z* 368.14908 (mass error = −0.169 ppm) and *m*/*z* 368.14905 (mass error = −0.199 ppm) (C_21_H_22_NO_5_), respectively. Both of them generated the same ESI-MS^2^ ions at *m*/*z* 353 [M − CH_3_]^+^, *m*/*z* 352 [M − CH_3_ − H]^+^, *m*/*z* 338 [M − 2CH_3_]^+^ and 324 [M − CH_3_ − H − CO]^+^. In addition, the ion at *m*/*z* 350 [M − H_2_O]^+^ suggested the presence of hydroxyl group at C-3 or C-13. Meanwhile, compound **55** displayed the ion [M − OCH_3_]^+^ at *m*/*z* 337, which indicated there were adjacent methoxyl and phenolic hydroxyl groups at the skeleton [23]. Therefore, the phenolic hydroxyl group of compound **19** and compound **55** existed at C-13 and C-3, respectively. Compound **19** and **55** were presumed to be 13-hydroxypalmatine and 3-hydroxy-2,4,9,10-tetramethoxy-5,6-dihydro-isoquino[3,2-*α*]isoquinolinylium.

Compound **42** showed the [M]^+^ ion at *m*/*z* 350.13852 (C_21_H_20_NO_4_, mass error = −0.470 ppm). It generated a serial of ions at *m*/*z* 335 [M − CH_3_]^+^, *m*/*z* 334 [M − CH_3_ − H]^+^, *m*/*z* 332 [M − H_2_O]^+^, *m*/*z* 320 [M − 2CH_3_]^+^ and *m*/*z* 306 [M − CH_3_ − H − CO]^+^. Thus, compound **42** was tentatively determined as 13-methylberberine.

Compound **9** and **13** generated [M]^+^ ions at *m*/*z* 356.18555 (mass error = −0.238 ppm) and *m*/*z* 356.18588 (mass error = 0.688 ppm) (C_21_H_26_NO_4_), respectively. The MS^2^ spectrum of compound **9** could produce the ion at *m*/*z* 311 [M − (CH_3_)_2_NH]^+^, which was the characteristic fragment ion of aporphine-type alkaloids [23]. The ion of compound **13** at *m*/*z* 340 was generated by loss a CH_4_ from the quasi-molecular ion. The ions at *m*/*z* 192 and *m*/*z* 165 were produced by Retro-Diels-Alder (RDA) cleavage fragmentation at 8, 13-position of the C-ring. Moreover, the product ion at *m*/*z* 192 generated the minor ion at *m*/*z* 190 and *m*/*z* 177 by the loss of two hydrogen ions and a methyl group, respectively. Therefore, according to the literature data, compounds **9** and **13** were tentatively deduced as menisperine and tetrahydropamatine [24].

Compound **2** gave a [M]^+^ ion at *m*/*z* 372.17999 (C_21_H_26_NO_5_, mass error = −1.503 ppm). The ion at *m*/*z* 354 [M − H_2_O]^+^ suggested the presence of C-3 or C-13 hydroxyl group. Meanwhile, the ion at *m*/*z* 356 [M − CH_3_ − H]^+^ indicated the presence of C9–C10 methoxyl groups [23]. The ion at *m*/*z* 192 [M + H − 181]^+^ was produced by RDA cleavage fragmentation at 8,13-position of the C-ring. The product ion at *m*/*z* 192 generated the minor ion at *m*/*z* 177 by the loss of a methyl group. Moreover, the ions at *m*/*z* 208 and *m*/*z* 165 were produced by α-cleavage fragmentation at B-ring. Therefore, compound **2** was tentatively presumed to be 13-hydroxy-2,3,9,10-tetramethoxy-5,8,13,13*a*-tetrahydro-6*H*-isoquino[3,2-*α*]isoquinolinium.

Compound **7** generated its [M]^+^ ion at *m*/*z* 356.18527 (C_21_H_26_NO_4_, mass error = −1.024 ppm). The ion at *m*/*z* 192 [M + H − 185]^+^ was yielded by RDA cleavage fragmentation at 8,13-position of the C-ring. Moreover, the product ion at *m*/*z* 192 generated the minor ions at *m*/*z* 190 and *m*/*z* 177 by the loss of two hydrogen ions and a methyl group, respectively. Moreover, the [M − OCH_3_]^+^ ions at *m*/*z* 325 indicated there were adjacent methoxyl and phenolic hydroxyl groups at the skeleton [23]. Therefore, compound **7** was tentatively deduced to be S-*trans*-N-methyltetrahydrocolumbamine*.*

Compound **15** generated its [M]^+^ ion at *m*/*z* 370.20117 (C_22_H_28_NO_4_, mass error = −0.310 ppm). It produced the ion at *m*/*z* 355, which involved the loss of a methyl group. The reaction of RDA cleavage took place in the course of which the characteristic ions at *m*/*z* 206 and *m*/*z* 165 are generated. In addition, the product ion at *m*/*z* 206 generated a serial of ions at *m*/*z* 204, *m*/*z* 191 and *m*/*z* 190 by the loss of two hydrogen ions, a methyl group and a molecule of methane, respectively. Therefore, compound **15** was tentatively determined as 2,3,9,10-tetramethoxy-7-methyl-5,8,13,13*a*-tetrahydro-6*H*-isoquino[3,2-*α*]isoquinolinium.

**Scheme 2:**
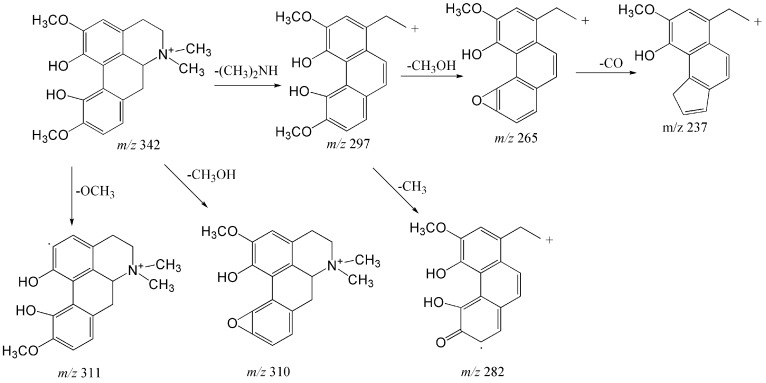
The proposed fragmentation pathway of magnoflorine.

Compounds **4** and **10** produced their [M]^+^ ions at *m*/*z* 342.16946 (mass error = −1.534 ppm) and *m*/*z* 342.17026 (mass error = 0.805 ppm) (C_20_H_24_NO_4_), respectively. Both of them generated the same ions at *m*/*z* 311 [M − OCH_3_]^+^. In the MS^2^ spectrum, the ion at *m*/*z* 192 which was generated by compound **10** suggested that the reaction of RDA cleavage took place. The further ions at *m*/*z* 190 [M – 150 − 2H]^+^ and 177 [M – 150 − CH_3_]^+^ indicated that compound **10** belonged to *N*-methyltetrahydroprotoberberine-type alkaloids [23]. However, compound **4** could generated the ESI-MS^2^ base peak ion at *m*/*z* 297, which involved the loss of a molecule of (CH_3_)_2_NH, a characteristic fragment ion of aporphine-type alkaloids. The proposed fragmentation pathway of compound **4** is shown in Scheme 2. Combined with bibliography data and fragmentation pathways, these two compounds were tentatively ascertained as magnoflorine and cyclanoline [25].

Compound **5** generated an [M − H + 2HCOOH]^−^ ion at *m*/*z* 414.15561 (C_22_H_24_NO_7_, mass error = 2.128 ppm). The ion at *m*/*z* 369 was produced by the loss of (CH_3_)_2_NH from the quasi-molecular ion, which suggested the compound **5** might be a kind of aporphine-type alkaloid [23].Moreover, it also generated fragments at *m*/*z* 386 [M − H + 2HCOOH − CO]^−^ and *m*/*z* 354 [M − H + 2HCOOH − CO − CH_3_OH]^−^. Therefore, compound **5** was tentatively determined as 2,11-dihydroxy-10-methoxy-6,6-dimethyl-5,6,6*a*,7-tetra-hydro-4*H*-dibenzo[*de,g*]quinolin-6-ium.

Compounds **1**, **3** and **11** produced their [M]^+^ ions at *m*/*z* 314.17465 (C_19_H_24_NO_3_, mass error = −1.337 ppm), 344.18536 (C_20_H_26_NO_4_, mass error = −0.798 ppm) and 328.19031 (C_20_H_26_NO_3_, mass error = −1.250 ppm), respectively. All of them generated [M − CH_3_]^+^, [M − CH_3_OH]^+^, [M − (CH_3_)_2_NH]^+^ and [M − (CH_3_)_2_NH − CH_3_OH]^+^ ion at *m*/*z* 299, *m*/*z* 282, *m*/*z* 269, *m*/*z* 237, and *m*/*z* 329, *m*/*z* 312, *m*/*z* 299, *m*/*z* 267, and *m*/*z* 313, *m*/*z* 296, *m*/*z* 283, *m*/*z* 251, respectively. The MS^2^ ions at *m*/*z* 175 (C_11_H_11_O_2_) were generated by *α*-cleavage fragmentation from 9,10-position of quasi-molecular ions. Compound **3** also yielded the ions at *m*/*z* 206 and *m*/*z* 137 through *α*-cleavage fragmentation at 8,9-position of [M]^+^ ion. Moreover, the product ion of compound **11** at *m*/*z* 175 generated the major ions at *m*/*z* 144 and *m*/*z* 143 by the loss of a methoxyl group and a molecule of methanol, respectively. Therefore, compound **1**, **3** and **11** were tentatively deduced as lotusine, tembetarine and colletine, respectively.

Compound **57** generated the [M + H]^+^ ion at *m*/*z* 294.11221 (C_18_H_16_NO_3_, mass error = −0.884 ppm). It generated a serial of ions at *m*/*z* 249 [M + H − CONH_3_]^+^, *m*/*z* 219 [M + H − CONH_3_ − CH_2_O]^+^, *m*/*z* 264 [M + H − CH_2_O]^+^, *m*/*z* 266 [M + H − CO]^+^ and *m*/*z* 236 [M + H − CO − CH_2_O]^+^. According to the fragmentation patterns, compound **57** was tentatively ascertained as N-formylannonain.

Compounds **48**, **53** and **54** generated their [M + H]^+^ ions at *m*/*z* 300.12320 (C_17_H_18_NO_4_, mass error = 0.551 ppm), *m*/*z* 284.12802 (C_17_H_18_NO_3_, mass error = −0.352 ppm) and *m*/*z* 314.13855 (C_18_H_20_NO_4_, mass error = −0.428 ppm), respectively. The ions at *m*/*z* 138 (C_8_H_12_NO) were generated by *α*-cleavage fragmentation at 9,10-position of [M + H]^+^ ion. The ESI-MS^2^ base peak ion of compound **54** at *m*/*z* 177 [M + 2H − C_8_H_12_NO]^+^ further generated [M + 2H − C_8_H_12_NO − CO]^+^, [M + 2H − C_8_H_12_NO − CH_3_OH]^+^ and [M + 2H − C_8_H_12_NO − CO − CH_3_OH]^+^ at *m*/*z* 149, *m*/*z* 145 and *m*/*z* 117, respectively. Similarly, the ESI-MS^2^ base peak ion of compound **48** at *m*/*z* 163 [M + 2H − C_8_H_12_NO]^+^ further generated [M + 2H − C_8_H_12_NO − CO]^+^ and [M + 2H − C_8_H_12_NO − C_2_H_2_O]^+^ at *m*/*z* 135 and 121, respectively. The ESI-MS^2^ base peak ion of compound **53** at *m*/*z* 147 [M + 2H − C_8_H_12_NO]^+^ further generated [M + 2H − C_8_H_12_NO − CO]^+^ at *m*/*z* 119. In addition, the ion of compound **53** at *m*/*z* 138 could generate ion at *m*/*z* 121 by the loss of NH_3_. By comparison with reference standards, compound **48** and **54** were predicatively deduced as *N*-*trans*-caffeoyltyramine and *N*-*trans*-feruloyltyramine. Compound **53** was tentatively identified as *N*-*p*-coumaroyltyramine.

#### 2.1.2. Structural Characterization and Identification of Phenylpropanoids

**Scheme 3:**
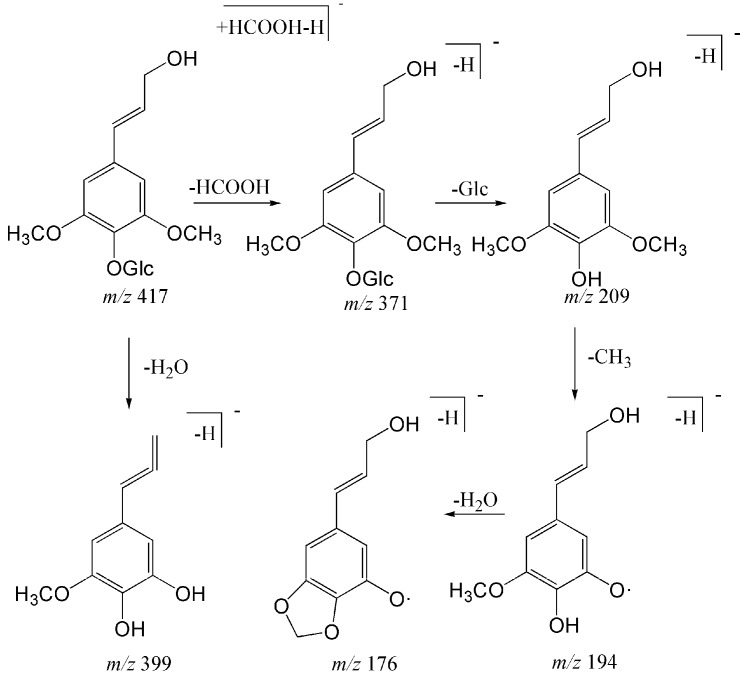
The proposed fragmentation pathway of *trans*-syringin.

Compound **6** produced the [M − H + HCOOH]^−^ ions at *m*/*z* 417.13998 (C_18_H_25_O_11_, mass error = 2.019 ppm). Its MS^2^ spectrum produced ions at *m*/*z* 399 and *m*/*z* 381, which involved the loss of one and two molecules of H_2_O, respectively. Moreover, the deprotonated molecular ions yield [M − H + HCOOH − CH_2_O]^−^ at *m*/*z* 387, [M − H + HCOOH − CO_2_]^−^ at *m*/*z* 373. In addition, compound **6** also produced a serial of ions at *m*/*z* 371 [M − H]^−^, *m*/*z* 209 [M − H − Glc]^−^, *m*/*z* 191 [M − H − Glc − H_2_O]^−^. The proposed fragmentation pathway of compound **6** is shown in Scheme 3. Compared with the *t*_R_ values and mass spectra with the reference standard, compound **6** was predicatively characterized as *trans*-syringin.

Compound **8** generated [M + Na]^+^ ion at *m*/*z* 527.17303 (C_22_H_32_O_13_Na, mass error = −0.914 ppm). The [M + Na]^+^ ion produced the ions at *m*/*z* 395 and *m*/*z* 233 in the MS^2^ spectrum, which originated from the neutral loss of an apiose moiety and a disaccharide moiety, which was composed of one molecule of glucose and one molecule of apiose. In addition, the molecular ion also produced the minor ion at *m*/*z* 496 [M + Na − OCH_3_]^+^. Thus, compound **8** was tentatively determined as tinosinen.

Compounds **20** and **49** yielded their [M + Na]^+^ ions at *m*/*z* 439.15729 (C_19_H_28_O_10_Na, mass error = −0.406 ppm) and *m*/*z* 481.16748 (C_21_H_30_O_11_Na, mass error = −1.149 ppm), respectively. Both of them generated [M + Na − Api]^+^ ions at *m*/*z* 307 and *m*/*z* 349, respectively. Compound **20** could generate a series of ions at *m*/*z* 421 [M + Na − H_2_O]^+^, *m*/*z* 403 [M + Na − 2H_2_O]^+^, *m*/*z* 407 [M + Na − CH_3_OH]^+^ and *m*/*z* 275 [M + Na − Api − H_2_O]^+^. Compound **49** displayed fragment ions at *m*/*z* 466, *m*/*z* 440 and *m*/*z* 317 corresponding to [M + Na − CH_3_]^+^, [M + Na − C_3_H_5_]^+^ and [M + Na − Api − CH_3_OH]^+^, respectively. By comparing with the literature data, compound **49** and **20** were tentatively deduced as 4-allyl-2-methoxyphenyl-6-*O*-β-d-glucopyranosyl(6→1)-β-d-apiofuranoside and icariside D1 [26].

Compound **18** generated [M + Na]^+^ ion at *m*/*z* 705.23627 (C_32_H_42_O_16_Na, mass error = −0.335 ppm). The ions at *m*/*z* 687 and *m*/*z* 661 were yielded by neutral loss of H_2_O and CO_2_, respectively. Moreover, the molecular ion generated the major ions at *m*/*z* 543 [M + Na − Glc]^+^, *m*/*z* 381 [M + Na − 2Glc]^+^ and *m*/*z* 528 [M + Na − Glc − CH_3_]^+^. According to the literature data, compound **18** was ascertained as pinoresinol-di-O-β-d-glucopyranoside [27].

Compounds **39** and **45** produced their [M + Na]^+^ ion at *m*/*z* 603.20416 (mass error = −1.081 ppm) and *m*/*z* 603.20441 (mass error = −0.667 ppm) (C_28_H_36_O_13_Na). Both of the molecular ions generated [M + Na − H_2_O]^+^, [M + Na − CH_3_]^+^, [M + Na − 2CH_3_]^+^, [M + Na − Glc]^+^, [M + Na − Glc − CH_3_]^+^ and [M + Na − Glc − H_2_O]^+^ at *m*/*z* 585, *m*/*z* 588, *m*/*z* 573, *m*/*z* 441, *m*/*z* 426 and *m*/*z* 423, respectively. In addition, compound **45** also yielded the [M + Na − CH_3_ − H]^+^ at *m*/*z* 587, which suggested the presence of adjacent methoxyl groups. Therefore, combined with literature data and fragmentation pathways, these two compounds were tentatively presumed to be syringaresinol-*O*-β-d-glucopyranoside and 2-{4-[4-(3-hydroxy-4,5-dimethoxyphenyl)-tetrahydrofuro[3,4-*c*]furan-1-yl]-2,6-dimethoxyphenoxy}-6-hydroxymethyltetra-hydropyran-3,4,5-triol, respectively [27].

Compound **43** produced [M + Na]^+^ ion at *m*/*z* 543.18329 (C_26_H_32_O_11_Na, mass error = −0.723 ppm). The ESI-MS^2^ base peak ion was [C_13_H_18_O_7_ + Na]^+^ at *m*/*z* 309. Moreover, it also generated ions at *m*/*z* 525 [M + Na − H_2_O]^+^, *m*/*z* 528 [M + Na − CH_3_]^+^, *m*/*z* 513 [M + Na − 2CH_3_]^+^, *m*/*z* 512 [M + Na − OCH_3_]^+^, *m*/*z* 381 [M + Na − Glc]^+^ and *m*/*z* 363 [M + Na − Glc − H_2_O]^+^. In addition, the distinctive ion at *m*/*z* 527 [M + Na − CH_3_ − H]^+^ indicated the presence of adjacent methoxyl groups. Thus, compound **43** was tentatively determined as 2-{4-[4-(3,4-dimethoxyphenyl)-tetrahydrofuro[3,4-*c*]furan-1-yl]-2-hydroxyphenoxy}-6-hydroxy-methyltetrahydropyran-3,4,5-triol.

Compound **23** generated [M − H + HCOOH]^−^ ion at *m*/*z* 787.26581 (C_35_H_47_O_20_, mass error = 0.368 ppm). It produced a serial of ions at *m*/*z* 769 [M − H + HCOOH − H_2_O]^−^, *m*/*z* 741 [M − H]^−^, *m*/*z* 23 [M − H − H_2_O]^−^, *m*/*z* 579 [M − H + HCOOH − Glc]^−^ and *m*/*z* 417 [M − H + HCOOH − 2Glc]^−^. According to the literature data, compound **34** was deduced as syringaresinol-di-*O*-β-d-glucopyranoside [27].

Compound **37** showed its [M − H]^−^ ion at *m*/*z* 519.18640 (C_26_H_31_O_11_, mass error = 0.601 ppm). The base peak ion at *m*/*z* 357 was originated from the neutral loss of a glucose moiety. It further generated the fragment ions at *m*/*z* 342 and *m*/*z* 339 by the loss of a molecule of methyl and H_2_O, respectively. In addition, it also could produce [M − H − H_2_O]^−^ at *m*/*z* 501. According to the literature data, it was tentatively ascertained as pinoresinol-*O*-β-d-glucopyranoside [27].

Compounds **40** and **52** produced their [M − H + HCOOH]^−^ ions at *m*/*z* 579.20740 (mass error = 0.315 ppm) and *m*/*z* 579.20703 (mass error = −0.324 ppm) (C_28_H_35_O_13_). Both of them produced [M − H + HCOOH − Glc]^−^ at *m*/*z* 417. Compound **52** generated a serial of ions at *m*/*z* 564 [M − H + HCOOH − CH_3_]^−^ and *m*/*z* 402 [M − H + HCOOH − Glc − CH_3_]^−^. Compound **40** could produce [M − H + HCOOH − H_2_O]^−^, [M − H + HCOOH − Glc − H_2_O]^−^, [M − H]^−^ and [M − H − Glc]^−^ at *m*/*z* 561, *m*/*z* 399, *m*/*z* 533 and *m*/*z* 371, respectively. In addition, compound **40** also generated the ion at *m*/*z* 563 [M − H + HCOOH − CH_4_]^−^, which suggested the presence of adjacent methoxyl groups. Therefore, according to the fragmentation pathways and literature data, compound **40** and **52** was tentatively presumed to be 2-{4-[4-(3,5-dimethoxyphenyl)-tetrahydrofuro[3,4-*c*]furan-1-yl]-2-methoxyphenoxy}-6-hydroxymethyltetrahydropyran-3,4,5-triol and pinoresinol monomethyl ether-*O*-β-d-glucopyranoside [27]. 

**Scheme 4:**
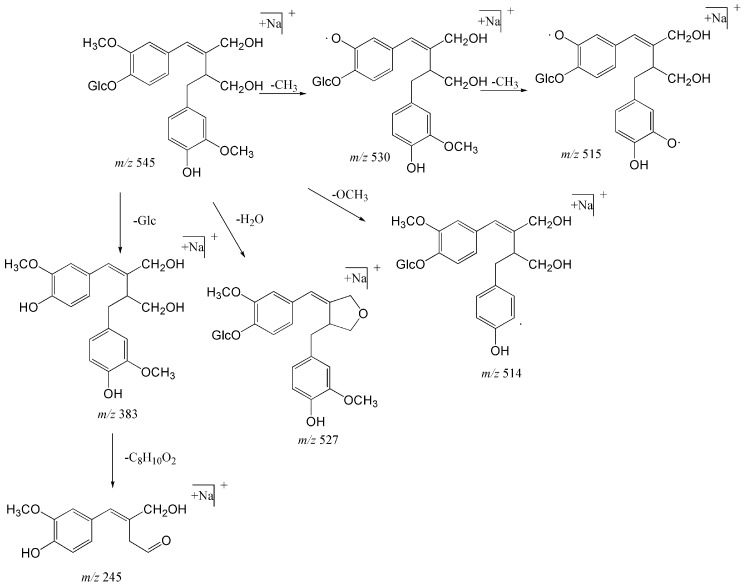
The proposed fragmentation pathway of sagitiside A.

Compounds **29** and **56** generated their [M + Na]^+^ ions at *m*/*z* 545.19855 (C_26_H_34_O_11_Na, mass error = −1.436 ppm) and *m*/*z* 383.14645 (C_20_H_24_O_6_Na, mass error = −0.156 ppm), respectively. The [M + Na]^+^ ion of compound **29** produced the aglycone ion at *m*/*z* 383 in the MS^2^ spectrum, which originated from the neutral loss of an glucose moiety (162 Da). Both of them could generate the same MS^2^ ions at *m*/*z* 245, which produced by a loss of neutral fragment (C_8_H_10_O_2_) from the ion at *m*/*z* 383. In addition, compound **29** generated a serial of ions at *m*/*z* 530 [M + Na − CH_3_]^+^, *m*/*z* 527 [M + Na − H_2_O]^+^, *m*/*z* 515 [M + Na − 2CH_3_]^+^, *m*/*z* 514 [M + Na − OCH_3_]^+^. Compound **56** generated [M + Na − CH_3_]^+^, [M + Na − H_2_O]^+^, [M + Na − CH_3_OH]^+^ and [M + Na − 2H_2_O]^+^ at *m*/*z* 368, *m*/*z* 365, *m*/*z* 351 and *m*/*z* 347, respectively. The proposed fragmentation pathway of compound **29** is shown in Scheme 4. Comparing with the literature data and respective fragmentation pathways, compound **29** and **56** was plausibly described as sagitiside A and 2-(4-hydroxy-3-methoxybenzyl)-3-(4-hydroxy-3-methoxybenzylidene)butane-1,4-diol.

Compound **46** produced its [M − H]^−^ ion at *m*/*z* 535.21783 (C_27_H_35_O_11_, mass error = 0.825 ppm). Its ESI-MS^2^ base peak ion at *m*/*z* 373 was generated by losing dehydrated glucose, and the major product ions at *m*/*z* 520 and *m*/*z* 505 were produced by the loss of one and two molecules of methyl from the quasi-molecular ion, respectively. In addition, the minor ion at *m*/*z* 357 [M − H − Glc − CH_4_]^−^ generated from the ion at *m*/*z* 373 suggested the presence of adjacent methoxyl groups. By comparison with reference standard and literature data, compound **46** was proposed to be tinosposide A [28].

Compound **25** and **36** showed their [M + Na]^+^ ions at *m*/*z* 547.21509 (mass error = 0.107 ppm) and *m*/*z* 547.21448 (mass error = −0.919 ppm) (C_26_H_36_O_11_Na). Both of their molecular ions generated a series of ions at *m*/*z* 532 [M + Na − CH_3_]^+^, *m*/*z* 529 [M + Na − H_2_O]^+^, *m*/*z* 517 [M + Na − CH_3_]^+^, *m*/*z* 514 [M + Na − CH_3_ − H_2_O]^+^, *m*/*z* 385 [M + Na − Glc]^+^, *m*/*z* 367 [M + Na − Glc − H_2_O]^+^ and *m*/*z* 349 [M + Na − Glc − 2H_2_O]^+^. Moreover, compound **25** could also produce ions at *m*/*z* 531 and *m*/*z* 383 by the loss of CH_4_ and neutral fragment (C_9_H_12_O_2_), respectively. It suggested that the presence of adjacent methoxyl groups. Therefore, according to the fragmentation pathways, compound **25** and **36** were deduced as 2-{4-[4-(3,4-dimethoxyphenyl)-2,3-bishydroxymethylbutyl]-2-hydroxyphenoxy}-6-hydroxymethyltetrahydro-pyran-3,4,5-triol and 2-{4-[4-(4-hydroxy-3-methoxyphenyl)-2,3-bishydroxymethylbutyl]-2-methoxy-phenoxy}-6-hydroxymethyl-tetrahydropyran-3,4,5-triol, respectively.

Compound **12** produced its [M − H + HCOOH]^−^ ion at *m*/*z* 583.20276 (C_27_H_35_O_14_, mass error = 1.077 ppm). As the ESI-MS^2^ base peak, the dominant characteristic ion was [M − H − Glc]^−^ at *m*/*z* 375, corresponding to the cleavage from the dehydrated glucose. The major ions at *m*/*z* 568 and *m*/*z* 565 were generated by the neutral loss of CH_3_ and H_2_O, respectively. In addition, the molecular ion also produced ions at *m*/*z* 537 [M − H]^−^, *m*/*z* 522 [M − H − CH_3_]^−^, *m*/*z* 357 [M − H − Glc − H_2_O]^−^ and *m*/*z* 327 [M − H − Glc − H_2_O − 2CH_3_]^−^. According to the literature data, it was identified as tanegoside [28].

Compounds **30** and **31** generated their [M + Na]^+^ ions at *m*/*z* 399.14093 (mass error = −1.238 ppm) and *m*/*z* 399.14133 (mass error = −0.236 ppm) (C_20_H_24_O_7_Na), respectively. The ESI-MS^2^ base peak ion at *m*/*z* 202 (C_10_H_11_O_3_Na) was produced by *α*-cleavage fragmentation from 8,12-position and 10,11-position of the B-ring. In addition, another major ion at *m*/*z* 219 (C_10_H_12_O_4_Na) was produced by *α*-cleavage fragmentation from 8,12-position and 9,10-position of the B-ring. Moreover, both of the molecular ions generated a serial of ions at *m*/*z* 381 [M + Na − H_2_O]^+^, *m*/*z* 368 [M + Na − CH_2_OH]^+^, *m*/*z* 384 [M + Na − CH_3_]^+^, *m*/*z* 369 [M + Na − 2CH_3_]^+^ and *m*/*z* 351 [M + Na − 2CH_3_ − H_2_O]^+^. According to the fragmentation pathways and the values of Clog *P*, compound **30** and **31** were tentatively determined as 8’-epitanegool and 4-[5-(4-hydroxy-3-methoxyphenyl)-4-hydroxymethyltetrahydrofuran-3-ylmethyl]-6-methoxybenzene-1,3-diol.

Compound **41** produced the [M + Na]^+^ ion at *m*/*z* 605.22021 (C_28_H_38_O_13_Na, mass error = −0.252 ppm). It generated its ESI-MS^2^ base peak ion at *m*/*z* 443 by loss of a dehydrated glucose, and further generated a serial of ions at *m*/*z* 425 [M + Na − H_2_O]^+^, *m*/*z* 413 [M + Na − 2CH_3_]^+^, *m*/*z* 412 [M + Na − OCH_3_]^+^ and *m*/*z* 395 [M + Na − H_2_O − 2CH_3_]^+^. In addition, the molecular ion also generated [M + Na − CH_3_]^+^, [M + Na − H_2_O]^+^ and [M + Na − OCH_3_]^+^ at *m*/*z* 590, *m*/*z* 578 and *m*/*z* 574, respectively. Combined with literature data, compound **41** was identified as lyoniresinol-2*α*-*O*-β-d-glucopyranoside [29].

Compounds **22** and **28** generated their [M − H]^−^ ions at *m*/*z* 359.14923 (mass error = 0.315 ppm) and *m*/*z* 359.14935 (mass error = 0.435 ppm) (C_20_H_23_O_6_), respectively. Both of their deprotonated molecular ions produced [M − H − CH_3_]^−^, [M − H − H_2_O]^−^, [M − H − 2CH_3_]^−^ and [M − H − 2CH_3_ − H_2_O]^−^ at *m*/*z* 344, *m*/*z* 341, *m*/*z* 329 and *m*/*z* 311, respectively. In addition, compound **22** could generate the ion at *m*/*z* 205 (C_12_H_13_O_3_) by losing a neutral fragment (C_8_H_10_O_3_). However, compound **28** produced ions at *m*/*z* 343 [M − H − CH_4_]^−^ and *m*/*z* 191 [M − H − C_9_H_12_O_3_]^−^. That indicated the presence of adjacent methoxyl groups of the C-ring. According to the fragmentation pathways and the values of Clog *P*, compound **22** and **28** were tentatively ascertained as 3(*a*,4-dihydroxy-3-methoxybenzyl)-4-(4-hydroxy-3-methoxybenzyl)tetrahydrofuran and 4-{4-[(3,4-dimethoxyphenyl)-hydroxymethyl]-tetrahydro-furan-3-ylmethyl}-benzene-1,2-diol.

#### 2.1.3. Structural Characterization and Identification of Sesquiterpenoids

Compounds **35**, **44** and **50** generated their [M + Na]^+^ ions at *m*/*z* 581.2564 (C_27_H_42_O_12_Na, mass error = −0.753 ppm), *m*/*z* 551.24579 (C_26_H_40_O_11_Na, mass error = −0.895 ppm) and *m*/*z* 419.20309 (C_21_H_32_O_7_Na, mass error = −2.229 ppm). That the deviation of compound **35** and **50** was 162 Da suggested compound **35** had one more glucose unit than compound **50**. Similarly, that the deviation of compound **44** and **50** was 132 Da suggested compound **44** had one more apiose unit than compound **50**. All of them displayed the same aglycone ions at *m*/*z* 257 (C_15_H_22_O_2_Na). In addition, compound **35** generated a serial of ions at *m*/*z* 563 [M + Na − H_2_O]^+^, *m*/*z* 551 [M + Na − 2CH_3_]^+^, *m*/*z* 533 [M + Na − 2CH_3_ − H_2_O]^+^, *m*/*z* 419 [M + Na − Glc]^+^, *m*/*z* 401 [M + Na − Glc − H_2_O]^+^, *m*/*z* 365 [M + Na − Glc − 3H_2_O]^+^ and *m*/*z* 347 [M + Na − Glc − 4H_2_O]^+^. Compound **44** generated a series of ions at *m*/*z* 533 [M + Na − H_2_O]^+^, *m*/*z* 521 [M + Na − 2CH_3_]+, *m*/*z* 503 [M + Na − 2CH_3_ − H_2_O]^+^, *m*/*z* 419 [M + Na − Api]^+^, *m*/*z* 387 [M + Na − Api − CH_3_OH]+, *m*/*z* 335 [M + Na − Api − C_5_H_8_O]^+^ and *m*/*z* 203 [M + Na − Api − Glc − C_3_H_2_O]^+^. Compared with the *t*_R_ values and mass spectra with the reference standard, compound **50** was predicatively identified as tinocordiside [30]. Compound **35** and **44** were tentatively presumed to be 6-{1-[3,4-dihydroxy-6-hydroxymethyl-5-(3,4,5-trihydroxy-6-hydroxymethyltetrahydropyran-2-yloxy)-tetrahydropyran-2-yloxy]-1-methylethyl}-2,12-dimethyltricyclo[6.4.0.0^2,9^]dodec-11-en-10-one and tinosinenside.

Compound **47** produced its [M − H + HCOOH]^+^ ion at *m*/*z* 543.20740 (C_25_H_35_O_13_, mass error = 0.336 ppm). It generated a series of ions at *m*/*z* 528 [M − H + HCOOH − CH_3_]^−^, *m*/*z* 525 [M − H + HCOOH − H_2_O]^−^, *m*/*z* 507 [M − H + HCOOH − 2H_2_O]^−^, *m*/*z* 497 [M − H + HCOOH − CH_3_ − CH_2_OH]^−^ and *m*/*z* 411 [M − H + HCOOH − Xyl]^−^. Therefore, it was tentatively identified as 3,9-dihydorxymegastigmane-3-*O*-β-d-gluco-pyranosyl(6→1)-β-d-xylpyranoside.

Compounds **32** and **38** produced [M + Na]^+^ ions at *m*/*z* 435.19821 (C_21_H_32_O_8_Na, mass error = −1.675 ppm) and *m*/*z* 273.14627 (C_15_H_22_O_3_Na, mass error = 0.565 ppm). The [M + Na]^+^ ion of compound **32** produced the aglycone ion at *m*/*z* 273 in the MS^2^ spectrum, which originated from the neutral loss of an glucose moiety (162 Da). Compound **38** produced a serial of ions at *m*/*z* 255 [M + Na − H_2_O]^+^, *m*/*z* 245 [M + Na − CO]^+^, *m*/*z* 230 [M + Na − CO − CH_3_]^+^ and *m*/*z* 227 [M + Na − H_2_O − CO]^+^. Compound **32** generated [M + Na − Glc]^+^, [M + Na − Glc − CO]^+^, [M + Na − Glc − H_2_O]^+^, [M + Na − H_2_O]^+^ and [M + Na − CO]^+^ at *m*/*z* 273, *m*/*z* 245, *m*/*z* 255, *m*/*z* 417 and *m*/*z* 407, respectively. According to the literature data, compound **32** and **38** were tentatively determined as tinocordifolioside and tinocordifolin [31].

Compound **51** generated its [M + Na]^+^ ion at *m*/*z* 487.21439 (C_21_H_36_O_11_Na, mass error = −1.217 ppm). It produced the [M + Na − Api]^+^ and [M + Na − Api − Glc]^+^ ions at *m*/*z* 355 and *m*/*z* 193 due to the overall fracture of dehydrated apiose and glucose. In addition, the molecular ion also produced a serial of ions at *m*/*z* 469 [M + Na − H_2_O]^+^, *m*/*z* 472 [M + Na − CH_3_]^+^, *m*/*z* 457 [M + Na − 2CH_3_]^+^ and *m*/*z* 439 [M + Na − 2CH_3_ − H_2_O]^+^. Therefore, compound **51** was tentatively deduced as angelicoidenol-2-*O*-β-d-apiofuranosyl-(1→6)-β-d-glucopyranoside.

#### 2.1.4. Structural Characterization and Identification of Other Compounds

Compound **60** showed [M − H + HCOOH]^−^ ion at *m*/*z* 621.43665 (C_36_H_61_O_8_Na, mass error = 0.893 ppm). Its ESI-MS^2^ base peak ion at *m*/*z* 575 was generated by losing CH_3_ and CO. In addition, the major ions at *m*/*z* 606 and *m*/*z* 603 were produced by neutral loss of H_2_O and CH_3_, respectively. Moreover, the molecular ion also yielded ions at *m*/*z* 585 [M − H + HCOOH − 2H_2_O]^−^ and *m*/*z* 560 [M − H + HCOOH − H_2_O − CO − CH_3_]^−^. By comparing with the literature data, compound **60** was tentatively identified as daucosterol [32].

Compound **58** and **59** produced their [M − H]^−^ ions at *m*/*z* 455.35257 (mass error = 1.314 ppm) and *m*/*z* 455.35291 (mass error = 2.060 ppm) (C_30_H_47_O_3_). Both of the deprotonated molecular ions yield [M − H − H_2_O]^−^, [M − H − 2H_2_O]^−^, [M − H − H_2_O − CH_2_O]^−^, [M − H − COOH]^−^ and [M − H − CO_2_]^−^ at *m*/*z* 437, *m*/*z* 419, *m*/*z* 407, *m*/*z* 410 and *m*/*z* 411. According to the literature data and the values of Clog *P*, compounds **58** and **59** were tentatively deduced as ursolic acid and oleanic acid [33].

### 2.2. Isolated Compounds Identification

The raw spectral analysis data of three compounds, which were purified and identified from *T. sinensis*, are listed below.

*Tinosinen* (**8**): C_22_H_32_O_13_, white powder. ESI-MS *m*/*z* 572.2 [M + Na]^+^ and *m*/*z* 543.1 [M + K]^+^. ^13^C-NMR (CD_3_OD,150 MHz) δ 135.3 (C-1), 154.3 (C-2,6), 105.4 (C-3,5), 135.8 (C-4), 131.3 (C-7), 130.1 (C-8), 63.6 (C-9), 57.0 (OCH3), 105.1 (C-1′), 75.5 (C-2′), 85.2 (C-3′), 69.8 (C-4′), 78.1 (C-5′), 62.5 (C-6′), 111.4 (C-1″), 77.9 (C-2″), 80.6 (C-3″), 75.0 (C-4″), 65.2 (C-5″). ^1^H-NMR (CD_3_OD, 600 MHz) δ 6.74 (2H, s, H-3, H-5), 6.53 (1H, d, *J* = 15.9 Hz, H-7), 6.31 (1H, dt, *J* = 15.9, 5.6 Hz, H-8), 4.21 (2H, d, *J* = 5.6 Hz, H-9), 3.84 (6H, s, OCH_3_), 4.89 (1H, d, *J* = 7.7 Hz, H-1′), 3.59 (1H, dd, *J* = 8.9, 7.7 Hz, H-2′), 3.51 (1H, dd, *J* = 8.9, 8.8 Hz, H-3′), 3.44 (1H, dd, *J* = 8.8, 9.5 Hz, H-4′), 3.22 (1H, m, H-5′), 3.65 (1H, dd, *J* = 12.1, 5.0 Hz, H-6′a), 3.78 (1H, m, H-6′b), 5.30 (1H, d, *J* = 2.7 Hz, H-1″), 4.00 (1H, d, *J* = 2.7 Hz, H-2″), 3.77 (1H, m, H-4″a), 4.11 (1H, d, *J* = 9.3 Hz, H-4″b), 3.60 (2H, s, H-5″). Compared with the literature data, tinosinen was confirmed [28].

*Syringaresinol*-*4*-*O*-β-*d*-*glucopyranoside* (**39**): C_28_H_36_O_13_, white powder. ESI-MS *m*/*z* 603.2 [M + Na]^+^. ^13^C-NMR (CD_3_OD, 125 MHz) δ 139.6 (C-1), 105.4 (C-2, 6), 154.4 (C-3, 5), 135.8 (C-4), 87.2 (C-7), 55.5 (C-8), 73.0 (C-9), 133.2 (C-1′), 104.8 (C-2′, 6′), 149.4 (C-3′, 5′), 136.4 (C-4′), 87.6 (C-7′), 55.7 (C-8′), 72.9 (C-9′), 57.2 (3, 5-OCH_3_), 56.9 (3′, 5′-OCH_3_), 105.0 (C-1″), 75.8 (C-2″), 77.8 (C-3″), 71.4 (C-4″), 78.3 (C-5″), 62.7 (C-6″). ^1^H-NMR (CD_3_OD, 500 MHz) δ 6.71 (2H, s, H-2, H-6), 4.71 (1H, d, *J* = 4.4 Hz, H-7), 3.13 (1H, m, H-8), 3.91 (1H, dd, *J* = 9.5, 3.2 Hz, H-9a), 4.30~4.26 (1H, m, H-9b), 6.65 (2H, s, H-2′, H-6′), 4.76 (1H, d, *J* = 4.1 Hz, H-7′), 3.13 (1H, m, H-8′), 3.91 (1H, dd, *J* = 9.5, 3.2 Hz, H-9′a), 4.30~4.26 (1H, m, H-9′b), 3.85 (6H, s, 3, 5-OCH_3_), 3.84 (6H, s, 3′, 5′-OCH_3_), 4.84 (1H, d, *J* = 7.7 Hz, H-1″), 3.48 (1H, m, H-2″), 3.42 (1H, m, H-3″), 3.41 (1H, m, H-4″), 3.20 (1H, m, H-5″), 3.66 (1H, dd, *J* = 11.9, 5.2 Hz, H-6″a), 3.76 (1H, m, H-6″b). Compared with the literature data, syringaresinol-4-*O*-β-d-glucopyranoside was confirmed [28].

*N*-*trans*-*Caffeoyltryramine* (**48**): C_17_H_17_O_4_N, white powder. ESI-MS *m*/*z* 298.1 [M − H]^−^. ^13^C-NMR (CD_3_OD, 125 MHz) δ 126.9 (C-1), 113.6 (C-2), 145.3 (C-3), 147.3 (C-4), 115.0 (C-5), 120.6 (C-6), 140.7 (C-7), 116.9 (C-8), 167.8 (C-9), 129.9 (C-1′), 129.3 (C-2′, C-6′), 114.8 (C-3′, C-5′), 155.5 (C-4′), 34.4 (C-7′), 41.1 (C-8′). ^1^H-NMR (CD_3_OD, 500 MHz) δ 6.97 (1H, d, *J* = 1.8 Hz, H-2), 6.74 (1H, d, *J* = 8.1 Hz, H-5), 6.88 (1H, dd, *J* = 8.1, 1.8 Hz, H-6), 7.36 (1H, d, *J* = 15.6 Hz, H-7), 6.31 (1H, d, *J* = 15.7 Hz, H-8), 7.03 (2H, d, *J* = 8.2 Hz, H-2′, H-6′), 6.70 (2H, d, *J* = 8.2 Hz, H-3′, H-5′), 2.73 (2H, t, *J* = 7.4 Hz, H-7′), 3.43 (2H, t, *J* = 7.4 Hz, H-8′). Compared with the literature data, *N*-*trans*-caffeoyltryramine was confirmed [34].

## 3. Materials and Methods

### 3.1. Materials and Chemicals

*T. Sinensis* was purchased from Anguo Linshi Medicinal Materials Co., Ltd. (Anguo, Hebei, China) and then authenticated by Professor Chun-sheng Liu, Beijing University of Chinese Medicine. Reference compounds, including *trans*-syringin, tinocordiside, tinosposide A, N-*trans*-caffeoyltyramine were isolated from *T. sinensis* by the authors and their structures were fully characterized by chemical and spectroscopic methods (NMR and MS). *N*-*trans*-feruloyltyramine and berberine were purchased from Shanghai Tauto Biotech CO., Ltd. (Shanghai, China). Formic acid, methanol and acetonitrile (HPLC grade) were purchased from Fisher Scientific (Fair Lawn, NJ, USA). Ultrapure water was purchased from Hangzhou Wahaha Group Co., Ltd. (Hangzhou, Zhejiang, China).

### 3.2. Sample and Standards Preparation

The standard solutions of *trans*-syringin, tinocordiside, tinosposide A, *N*-*trans*-caffeoyltyramine, *N*-*trans*-feruloyltyramine and berberine were prepared in methanol at appropriate concentrations. Appropriate amounts of powdered dried alcoholic extracts of *T. sinensis*, which were refluxed with tenfold ethanol/water (70:30, *v*/*v*) for three times, were weighed precisely (0.13 g). The extracts were placed into 20 mL of methanol/water (70:30, *v*/*v*) and ultrasonically extracted at room temperature for 0.5 h, and then supplemented the loss with the same solvent. The mixture was filtrated and evaporated to nearly dry, and then washed it through Solid Phrase Extraction (SPE) C_18_ columns (J.T. Baker, Center Valley, PA, USA) with 4 mL distilled water and 4 mL methanol. Methanol eluent was filtered through a 0.22 µm membrane for analysis. All of the solutions were stored at 4 °C and brought to room temperature before analysis.

### 3.3. Extraction and Isolation

Ten kilogram of dried *T. sinensis* stems was extracted three times with reflux extraction method with 70% ethanol at 80 °C for 2 h. All extraction solutions were evaporated under reduced pressure to obtain the crude residue, which was suspended in pure water. Sequential liquid–liquid extraction for successive sample partition was performed by chloroform (CHCl_3_), ethyl acetate (EA), *n*-butanol (*n*-BuOH) and methyl alcohol (MeOH). The ethyl acetate (EA) extraction was subjected to silica gel columns with the step-gradient solvent system of petroleum ether (PE):ethyl acetate (EA) (12:1→0:1, *v*/*v*) and ethyl acetate (EA):methyl alcohol (MeOH) (6:1→0:1, *v*/*v*). And then Y90-101 was further isolated by multi silica gel columns, ODS column and Sephadex LH-20 column to yielded compound **48** (4.2 mg). The *n*-BuOH extraction was passed through an AB-8 macroporous resin column and then washed with H_2_O, 30% EtOH, 50% EtOH, 70% EtOH and 95% EtOH. The 30% EtOH fraction was further purified by Silica gel columns with elution of CHCl_3_–MeOH (15:1→0:1, *v*/*v*). Then, sample 11–23 was further isolated by Sephadex LH-20 column and multi silica gel columns to get compound **8** (3.2 mg) in CHCl_3_:MeOH:H_2_O (15:1:0.05). Sample 41–53 was further purified by multi silica gel columns to yield compound **39** (4.8 mg) in CHCl_3_:MeOH:H_2_O (15:1:0.05).

### 3.4. Instrumentation and Condition

HPLC analysis was performed on DIONEX Ultimate 3000 UHPLC system (Thermo Fisher Scientific, Waltham, MA, USA) with a binary pump and an autosampler. Samples were separated on a Sunfire C_18_ column (250 × 4.6 mm i.d., 5 μm, Waters Corporation, Milford, MA, USA) at room temperature. The mobile phase consisted of 0.1% (*v*/*v*) formic acid and acetonitrile (B). A gradient program was adopted as follows: 0–5 min, 8–12% B; 5–25 min, 12–16% B, 25–45 min, 16–25% B; 45–75 min, 25–46% B; 75–80 min, 46–58% B; 80–95 min, 58–65% B; 95–105 min, 65–68% B; 105–130 min, 68–95% B; 130–140 min, 95–92% B. The flow rate was set as 1.0 mL/min.

The LTQ-Orbitrap XL mass spectrometer (Thermo Scientific (Bremen), Bremen, Germany) was connected to the HPLC system *via* an electrospray ionization (ESI) interface in a post-column splitting ratio of 1:4. The analysis was performed in both negative and positive ion mode with a mass range of *m*/*z* 100–1500. The optimized ESI parameters in negative ion mode were set as follows: capillary temperature of 350 °C; sheath gas (nitrogen) flow of 30 arb.; auxiliary gas (nitrogen) flow of 10 arb.; source voltage of 4.0 kV; capillary voltage of −35 V; tube lens voltage of −110 V. The capillary voltage was 25 V and tube lens voltage was 110 V in positive ion mode; and other parameters were same as those of negative ion mode. The resolution of the orbitrap mass analyzer was set at 30,000. The isolation width was 2 amu, and the normalized collision energy (CE) was set to 35%. Collision-induced dissociation (CID) was conducted in LTQ with an activation *q* of 0.25 and activation time of 30 ms. All instruments were controlled by the Xcalibur data system, and the data acquisition was carried out by analyst software Xcalibur (version 2.1) (Waltham, MA, USA) from Thermo Electron Corp.

^1^H (500 MHz), ^13^C (125 MHz) spectra were recorded on a Bruker AVANCE-500 NMR spectrometer (Bruker Daltonik GmbH, Rheinstetten, Karlsruhe, Germany). ^1^H (600 MHz), ^13^C (150 MHz) spectra were recorded on a Varian Inova 600 spectrometer (Varian Medical Systems, Palo Alto, CA, USA).

## 4. Conclusions

In this study, an effective and sensitive analytical method by HPLC-LTQ-Orbitrap-MS^n^ was established for systematically characterizing non-diterpenoid constituents and guiding the extraction and isolation in *T. sinensis* extract. A total of 60 compounds attributed to four categories including 27 alkaloids, 23 phenylpropanoids, seven sesquiterpenoids and three other compounds were identified or tentatively characterized according to the *t*_R_ and fragmentation pathways. In previous work, we have established a method for the content determination of total alkaloids in *T. sinensis*, and the results demonstrated that the alkaloid constituents were abundant in this herb, which were in accordance with this study [35]. Besides, 20 compounds were firstly characterized in the genus *Tinospora* and 13 of them were new compounds. Three natural compounds, including two phenylpropanoids and an alkaloid, were purified and identified from *T. sinensis* by systemic separation, which also demonstrated that the results of HPLC-LTQ-Orbitrap-MS^n^ was reliable. The results serve well to illustrate the potential fragmentation pathways of non-deterpenoid constituents in *T. sinensis*, and the HPLC-LTQ-Orbitrap MS^n^ platform was proved as an effective tool for rapid qualitative analysis of constituents. This study not only provides abundant information for better understanding of the chemical compounds in *T. sinensis*, but also benefits further quality control of this medicine.

## Figures and Tables

**Table 1:** Identification of chemical constituents of *T. sinensis* by HPLC-LTQ-Orbitrap.

NO.	*t*_R_/min	Identification	Experical Formula	Proposal Ions	Theoretical Mass *m*/*z*	Experimental Mass *m*/*z*	Mass Error (ppm)	MS^2^ Data (Measured)
**1**	7.05	Lotusine ^b^	C_19_H_24_NO_3_	[M]^+^	314.17507	314.17465	−1.337	269 (100), 237 (8), 107 (12.85), 175 (15), 282 (4.7), 299(25)
**2**	8.02	13-Hydroxy-2,3,9,10-tetramethoxy-5,8,13,13*a*-tetrahydro-6*H*-isoquino[3,2-*α*]-isoquinolinium *^,b^	C_21_H_26_NO_5_	[M]^+^	372.18054	372.17999	−1.503	192 (100), 177 (3), 176 (0.1), 208 (29), 165 (0.15), 356 (0.61), 354 (1.79)
**3**	8.96	Tembetarine	C_20_H_26_NO_4_	[M]^+^	344.18563	344.18536	−0.798	299 (100), 175 (44), 137 (17), 267 (12), 312 (11), 206 (2), 329 (3)
**4**	9.41	Magnoflorine	C_20_H_24_NO_4_	[M]^+^	342.16998	342.16946	−1.534	297 (100), 265 (23), 311 (16), 310 (1.5), 282 (2.6), 237 (1)
**5**	11.02	2,11-dihydroxy-10-methoxy-6,6-dimethyl-5,6,6a,7-tetrahydro-4*H*-dibenzo[*de*,*g*]quinolin-6-ium *^,b^	C_22_H_24_NO_7_	[M − H + 2HCOOH]^−^	414.15472	414.15561	2.128	354 (100), 368 (42), 353 (30), 386 (12), 369 (0.3)
**6**	11.24	*Trans*-syringin ^a^	C_18_H_25_O_11_	[M − H + HCOOH]^−^	417.13913	417.13998	2.019	371 (3.3), 209 (100), 191 (10.62), 179 (12.32), 387 (4.76), 373 (10.54), 399 (13.63), 381 (7.27)
**7**	11.40	*S*-*trans*-*N*-methyltetra-hydrocolumbamine	C_21_H_26_NO_4_	[M]^+^	356.18563	356.18527	−1.024	192 (100), 177 (1.2), 190 (1), 325 (0.1)
**8**	13.40	Tinosinen	C_22_H_32_O_13_Na	[M + Na]^+^	527.17351	527.17303	−0.914	317 (100), 496 (7), 395 (3.8), 233 (3)
**9**	14.23	Menisperine	C_21_H_26_NO_4_	[M]^+^	356.18563	356.18555	−0.238	311 (100), 279 (74), 251 (1.8), 325 (6.6), 296 (7.1)
**10**	14.59	Cyclanoline	C_20_H_24_NO_4_	[M]^+^	342.16998	342.17026	0.805	192 (100), 190 (4), 177 (3.8), 311 (1.4)
**11**	15.63	Colletine ^b^	C_20_H_26_NO_3_	[M]^+^	328.19072	328.19031	−1.250	283 (100), 251 (8), 175 (19), 143 (4), 144 (1), 296 (5.6), 313 (1.7)
**12**	16.16	Tanegoside	C_27_H_35_O_14_	[M − H + HCOOH]^−^	583.20213	583.20276	1.077	375 (100), 537 (17.89), 357 (0.32), 327 (18.28), 568 (0.59), 565 (0.34), 522 (0.25)
**13**	18.02	Tetrahydropamatine	C_21_H_26_NO_4_	[M]^+^	356.18563	356.18588	0.688	192 (100), 177 (2.4), 190 (2), 165 (0.2), 340 (0.5)
**14**	18.97	Stepharanine	C_19_H_18_NO_4_	[M]^+^	324.12303	324.12292	−0.115	309 (100), 280 (2.2), 294 (0.34), 292 (0.16), 307 (3.2)
**15**	19.39	2,3,9,10-Tetramethoxy-7-methyl-5,8,13,13*a*-tetrahydro-6*H*-isoquino[3,2-*α*]-isoquinolinium *^,b^	C_22_H_28_NO_4_	[M]^+^	370.20128	370.20117	−0.310	206 (100), 204 (2.2), 191 (1.5), 190 (1.47), 165 (0.24), 355 (0.38)
**16**	19.95	Dehydrodiscretamine	C_19_H_18_NO_4_	[M]^+^	324.12303	324.12311	0.075	309 (100), 280 (1.2), 306 (0.17), 294 (0.08), 292 (0.05)
**17**	20.36	Demethyleneberberine	C_19_H_18_NO_4_	[M]^+^	324.12303	324.12277	−0.137	309 (100), 280 (11), 308 (9), 294 (0.8), 292 (0.56), 306 (0.22)
**18**	21.15	Pinoresinol-di-*O*-β-d-glucopyranoside ^b^	C_32_H_42_O_16_Na	[M + Na]^+^	705.23650	705.23627	−0.335	543 (100), 687 (1), 528 (0.58), 381 (0.16), 661 (0.16)
**19**	22.62	13-Hydroxypalmatine	C_21_H_22_NO_5_	[M]^+^	368.14924	368.14908	−0.169	353 (100), 352 (99), 350 (20), 324 (21), 334 (0.6), 338 (1)
**20**	24.27	Icariside D1	C_19_H_28_O_10_Na	[M + Na]^+^	439.15746	439.15729	−0.406	307 (100), 421 (2.6), 275 (1.6), 403 (0.16), 407 (0.24)
**21**	25.24	3-Hydroxy-2,9,11-trimethoxy-5,6-dihydro isoquino[3,2-*α*]-isoquinolinylium	C_20_H_20_NO_4_	[M]^+^	338.13868	338.13861	−0.075	323 (100), 295 (0.6), 308 (0.2), 294 (2), 320 (0.05)
**22**	26.15	3(*a*,4-dihydroxy-3-methoxybenzyl)-4-(4-*h*ydroxy-3-methoxybenzyl) tetrahydrofuran	C_20_H_23_O_6_	[M − H]^−^	359.14891	359.14923	0.315	341 (100), 344 (0.11), 329 (51), 311 (0.32), 205 (1.19)
**23**	26.55	Syringaresinol-di-*O*-β-d-glucoside ^b^	C_35_H_47_O_20_	[M − H + HCOOH]^−^	787.26552	787.26581	0.368	579 (100), 417 (14), 769( 12), 741 (14), 723 (4)
**24**	26.73	Palmaturbine	C_20_H_20_NO_4_	[M]^+^	338.13868	338.13837	−0.315	323 (100), 295 (0.5), 308 (0.1), 294(2)
**25**	27.41	2-{4-[4-(3,4-Dimethoxy-phenyl)-2,3-bis-hydroxymethylbutyl]-2-hydroxyphenoxy}-6-hydroxymethyl-tetrahydropyran-3,4,5-triol *,^b^	C_26_H_36_O_11_Na	[M + Na]^+^	547.21498	547.21509	0.107	532 (9.45), 385 (100), 367 (0.31), 349 (0.04), 514 (0.32), 517 (0.84), 529 (2.46), 531(1.5), 395(0.32)
**26**	27.98	Jatrorrhizine	C_20_H_20_NO_4_	[M]^+^	338.13868	338.13834	−0.345	323 (100), 322 (6), 295 (1), 308 (0.4), 294 (5), 320 (0.02)
**27**	29.04	Columbamine	C_20_H_20_NO_4_	[M]^+^	338.13868	338.13843	−0.255	323 (100), 322 (14), 308 (1.5), 295 (1), 294 (16)
**28**	31.17	4-{4-[(3,4-Dimethoxy-phenyl)hydroxymethyl]-tetrahydrofuran-3-ylmethyl}-benzene-1,2-diol *^,b^	C_20_H_23_O_6_	[M − H]^−^	359.14891	359.14935	0.435	341 (100), 344 (0.28), 329 (48), 311 (1.41), 191 (0.22), 343 (0.06)
**29**	31.50	Sagitiside A	C_26_H_34_O_11_Na	[M + Na]^+^	545.19933	545.19855	−1.436	530 (100), 383 (34), 527 (14.88), 515 (11.30), 514 (3.30), 245 (1.32)
**30**	31.97	8′-Epitanegool	C_20_H_24_O_7_Na	[M + Na]^+^	399.14142	399.14093	−1.238	202 (100), 351 (37), 381 (33), 384 (22), 368 (8), 369 (9), 219 (7.5)
**31**	33.68	4-[5-(4-Hydroxy-3-methoxyphenyl)-4-hydroxymethyl-tetrahydrofuran-3-ylmethyl]-6-methoxy-benzene-1,3-diol *^,b^	C_20_H_24_O_7_Na	[M + Na]^+^	399.14142	399.14133	−0.236	202 (100), 351 (27), 381 (19.7), 384 (24), 369 (12), 368 (4), 219 (8.3)
**32**	33.78	Tinocordifolioside	C_21_H_32_O_8_Na	[M + Na]^+^	435.19893	435.19821	−1.675	417 (20.57), 402 (0.13), 399 (0.78), 407 (8.28), 389 (1.93), 420 (0.53), 405 (0.77), 273 (100), 255 (2.15), 245 (0.15)
**33**	33.97	Berberine ^a^	C_20_H_18_NO_4_	[M]^+^	336.12303	336.12317	0.135	321 (100), 320 (25), 292 (21), 306 (1), 334 (0.66)
**34**	35.15	Palmatine	C_21_H_22_NO_4_	[M]^+^	352.15433	352.1539	−0.435	337 (69), 336 (100), 322 (0.5), 308 (20)
**35**	37.33	6-{1-[3,4-Dihydroxy-6-hydroxymethyl-5-(3,4,5-trihydroxy-6-hydroxy-methyl-tetrahydropyran-2-yloxy)-tetrahydro-pyran-2-yloxy]-1-methylethyl}-2,12-dimethyltricyclo-[6.4.0.02,9]-dodec-11-en-10-one *^,b^	C_27_H_42_O_12_Na	[M + Na]^+^	581.25684	581.25641	−0.753	365 (100), 347 (1.22), 563 (0.7), 551 (0.17), 533 (0.10), 419 (1.26), 401 (0.12), 257 (0.11)
**36**	37.77	2-{4-[4-(4-Hydroxy-3-methoxyphenyl)-2,3-bis-hydroxymethylbutyl]-2-methoxyphenoxy}-6-hydroxymethyl-tetrahydro-pyran-3,4,5-triol *,^b^	C_26_H_36_O_11_Na	[M + Na]^+^	547.21498	547.21448	−0.919	532 (100), 367 (42), 385 (35), 349 (8.6), 529 (13), 514 (5.8), 517 (2.5)
**37**	38.60	Pinoresinol-O-β-d-glucopyranoside	C_26_H_31_O_11_	[M−H]^−^	519.18608	519.1864	0.601	357 (100), 151 (0.21), 501 (0.04), 342 (0.08), 339 (0.02)
**38**	38.69	Tinocordifolin	C_15_H_22_O_3_Na	[M + Na]^+^	273.14611	273.14627	0.565	258 (11.24), 243 (10.32), 245 (27.57), 230 (4.6), 255 (100), 227 (27.93)
**39**	39.57	Syringaresinol-O-β-d-glucopyranoside	C_28_H_36_O_13_Na	[M + Na]^+^	603.20481	603.20416	−1.081	441 (100), 573 (8.88), 588 (6.6), 585 (1.8), 426 (0.49), 423 (0.28)
**40**	39.81	2-{4-[4-(3,5-Dimethoxy-phenyl)tetrahydro-furo[3,4-*c*]furan-1-yl]-2-methoxyphenoxy}-6-hydroxymethyl-tetrahydropyran-3,4,5-triol *^,b^	C_28_H_35_O_13_	[M − H + HCOOH]^−^	579.20721	579.20740	0.315	417 (100), 165 (0.08), 402 (0.08), 564 (0.04), 547 (0.02)
**41**	40.47	Lyoniresinol-2*α*-O-β-d-glucopyranoside	C_28_H_38_O_13_Na	[M + Na]^+^	605.22046	605.22021	−0.252	443 (100), 425 (2), 413 (10), 395 (40.88), 412 (2.62), 590 (1.71), 576 (1.14), 574 (15.34), 587 (8.55)
**42**	40.47	13-methylberberine ^b^	C_21_H_20_NO_4_	[M]^+^	350.13868	350.13852	−0.47	335 (100), 334 (99), 306 (39), 332 (0.85), 320 (0.56)
**43**	41.22	2-{4-[4-(3,4-Dimethoxy-phenyl)tetrahydro-furo[3,4-*c*]furan-1-yl]-2-hydroxyphenoxy}-6-hydroxymethyl-tetrahydropyran-3,4,5-triol *^,b^	C_26_H_32_O_11_Na	[M + Na]^+^	543.18368	543.18329	−0.723	309 (100), 381 (38), 527 (25), 528 (24), 525 (17), 363 (7.8), 513 (8), 512 (6.49)
**44**	41.23	Tinosinenside	C_26_H_40_O_11_Na	[M + Na]^+^	551.24628	551.24579	−0.895	335 (100), 503 (0.2), 521 (0.25), 533 (0.9), 419 (1.57), 387 (0.19), 257 (0.31), 239 (0.59), 203 (0.41)
**45**	41.43	2-{4-[4-(3-Hydroxy-4,5-dimethoxyphenyl)-tetrahydrofuro[3,4-*c*]-furan-1-yl]-2,6-dimethoxy-phenoxy}-6-hydroxymethyl-tetrahydro-pyran-3,4,5-triol *^,b^	C_28_H_36_O_13_Na	[M + Na]^+^	603.20481	603.20441	–0.667	441 (100), 573 (9.72), 588 (6.62), 585 (4.68), 426 (0.88), 423 (5.25), 587 (1)
**46**	41.91	Tinosposide A ^a^	C_27_H_35_O_11_	[M − H]^−^	535.21738	535.21783	0.825	373 (100), 520 (1.14), 358 (4.6), 517 (1.05), 505 (0.39), 357 (0.33), 358 (3.92)
**47**	41.97	3,9-Dihydroxy-megastigmane-3-*O*-β-d-glucopyranosyl(6→1)-β-d-xylpyranoside	C_25_H_35_O_13_	[M − H + HCOOH]^−^	543.20721	543.20740	0.336	528 (0.34), 497 (100), 525 (0.66), 507 (0.51), 411 (0.32)
**48**	41.99	N-*trans*-caffeoyltyramine ^a, b^	C_17_H_18_NO_4_	[M + H]^+^	300.12303	300.12320	0.551	163 (100), 138 (8.48), 135 (0.66), 121 (9.2)
**49**	42.29	4-allyl-2-methoxyphenyl-6-*O*-β-d-glucopyranosyl-(6→1)-β-d-apiofuranoside	C_21_H_30_O_11_Na	[M + Na]^+^	481.16803	481.16748	–1.149	349 (100), 317 (86), 440 (23), 291 (11), 466 (8)
**50**	43.42	Tinocordiside ^a^	C_21_H_32_O_7_Na	[M + Na]^+^	419.20402	419.20309	–2.229	401 (1.1), 391 (0.15), 375 (0.2), 257 (32.05), 239 (59.3), 203 (100), 185 (4.15)
**51**	43.80	Angelicoidenol-2-*O*-β-d-apiofuranosyl-(1→6)-β-d-glucopyranoside	C_21_H_36_O_11_Na	[M + Na]^+^	487.21498	487.21439	–1.217	469 (5.76), 472 (0.37), 457 (1.55), 439 (1.50), 355 (100), 337 (0.12), 193 (0.12)
**52**	49.24	Pinoresinol monomethyl ether-*O*-β-d-gluco-pyranoside	C_28_H_35_O_13_	[M − H + HCOOH]^−^	579.20721	579.20703	–0.324	563 (0.5), 371 (100), 533 (15.8), 417 (5.4), 561 (0.36), 399 (0.56)
**53**	49.78	*N*-*p*-Coumaroyltyramine ^b^	C_17_H_18_NO_3_	[M + H]^+^	284.12811	284.12802	–0.352	147 (100), 138 (0.18), 121 (0.94), 119 (1.87)
**54**	51.86	*N*-*trans*-feruloyltyramine ^a^	C_18_H_20_NO_4_	[M + H]^+^	314.13868	314.13855	–0.428	177 (100), 145 (7.8), 117 (0.89), 149 (0.14), 138 (0.02)
**55**	70.84	3-Hydroxy-2,4,9,10-tetramethoxy-5,6-dihydro-isoquino[3,2-*α*]-isoquinolinylium *^,b^	C_21_H_22_NO_5_	[M]^+^	368.14924	368.14905	–0.199	352 (100), 353 (85), 337 (62), 322 (27), 321 (2.4), 350 (1.5), 338 (12), 324 (17)
**56**	78.44	2-(4-Hydroxy-3-methoxy-benzyl)-3-(4-hydroxy-3-methoxy-benzylidene)-butane-1,4-diol *^,b^	C_20_H_24_O_6_Na	[M + Na]^+^	383.14650	383.14645	–0.156	365 (100), 368 (43), 351 (12), 347 (10), 245 (1)
**57**	80.17	N-Formylannonain	C_18_H_16_NO_3_	[M + H]^+^	294.11246	294.11221	–0.884	249 (100), 219 (3.2), 264 (7), 266 (0.85), 236 (0.8)
**58**	116.95	Ursolic acid	C_30_H_47_O_3_	[M − H]^−^	455.35197	455.35257	1.314	437 (21.06), 419 (4.9), 411 (45.93), 397 (13.61), 410 (14.24), 407 (19.45)
**59**	118.78	Oleanic acid	C_30_H_47_O_3_	[M − H]^−^	455.35197	455.35291	2.060	437 (1.65), 419 (1.63), 411 (4.66), 397 (5.75), 410 (2.13), 407 (100)
**60**	125.78	β-Sitosterol glycoside	C_36_H_61_O_8_	[M − H + HCOOH]^−^	621.43609	621.43665	0.893	575 (100), 560 (21), 603 (26.74), 585 (15), 606 (8.33)

^a^ Comparison with standards; ^b^ Firstly characterized in the genus *Tinospora*; * Tentatively identified as new compound.
